# Prevalence, prognosis, and treatment of atherosclerotic intracranial stenosis in Caucasians

**DOI:** 10.1177/1747493020974461

**Published:** 2020-12-03

**Authors:** Robert Hurford, Peter M Rothwell

**Affiliations:** Wolfson Centre for the Prevention of Stroke and Dementia, Nuffield Department of Clinical Neurosciences, 6396 University of Oxford, Oxford, UK

**Keywords:** Angiography, epidemiology, ischemic stroke, secondary prevention, stenosis, stroke prognosis

## Abstract

**Background:**

Intracranial atherosclerotic stenosis is a highly prevalent cause of stroke worldwide with important ethnic disparities. Widely considered to be a common cause of stroke in Asian and Afro-Caribbean populations, relatively less is known about the burden and significance of intracranial atherosclerotic stenosis in Caucasians.

**Aims:**

We aim to highlight recent insights and advances into the prevalence, prognosis, and treatment of symptomatic and asymptomatic atherosclerotic intracranial atherosclerotic stenosis in Caucasian patients.

**Summary of review:**

We identified 48 articles studying intracranial atherosclerotic stenosis in Caucasian patients with ischemic stroke or transient ischemic attack. Most studies were on hospital-based cohorts of consecutive patients and half were graded as “fair” quality. There was significant variation between studies in the definition of intracranial atherosclerotic stenosis and in the imaging modalities used to detect intracranial atherosclerotic stenosis. Overall, 12.1% of Caucasian patients were found to have any intracranial atherosclerotic stenosis, 6.4% symptomatic intracranial atherosclerotic stenosis and 11.1% asymptomatic intracranial atherosclerotic stenosis, with higher rates at older ages. In studies reporting prognosis, there were 61 and 10 same-territory ischemic strokes in 1000 person-years in patients with symptomatic and asymptomatic intracranial atherosclerotic stenosis, respectively. Percutaneous stenting and angioplasty have not proven superior to intensive medical management in patients with symptomatic intracranial atherosclerotic stenosis.

**Conclusions:**

Intracranial atherosclerotic stenosis has previously been neglected as a cause of stroke in Caucasians but is highly prevalent at older ages and frequently discovered with the growing use of noninvasive angiography. Intensive medical therapy is the treatment of choice, but there is a need to develop novel treatments or therapeutic approaches to lower the risk of stroke in higher risk patients.

## Introduction

Ischemic stroke is a heterogeneous disease and up to a fifth of cases are caused by atherosclerosis of the aortic arch, neck, or intracranial arteries.^1^ There is significant ethnic variation in the location of large artery disease; Asian, Hispanic, and Black populations have a high burden of intracranial atherosclerotic stenosis (ICS), accounting for a third of ischemic cerebrovascular events,^2–6^ whereas relatively less is known about the burden of ICS in Caucasians, in whom extracranial carotid artery atherosclerosis is considered predominant and ICS is only attributed to 5–10% of all ischemic strokes.^2,7^

Given the perceived lack of importance of ICS in Caucasians, routine screening for extracranial internal carotid artery stenosis is recommended by US and European guidelines but there is no consensus on the value of routine screening for ICS. Furthermore, the most appropriate screening modality with adequate sensitivity, specificity, and practicability remains contested.

ICS are more frequently detected by the increasing use of intracranial angiography in the assessment of acute stroke patients, posing a challenge to clinicians to accurately counsel patients about the likely prognosis and optimal treatment strategy. Although intensive medical therapy has been established as standard secondary prevention therapy by randomized trials,^8,9^ it remains to be seen whether risk of recurrent stroke can be further reduced by percutaneous angioplasty and stenting or novel surgical approaches in high-risk subgroups of patients.

**Figure 1.**
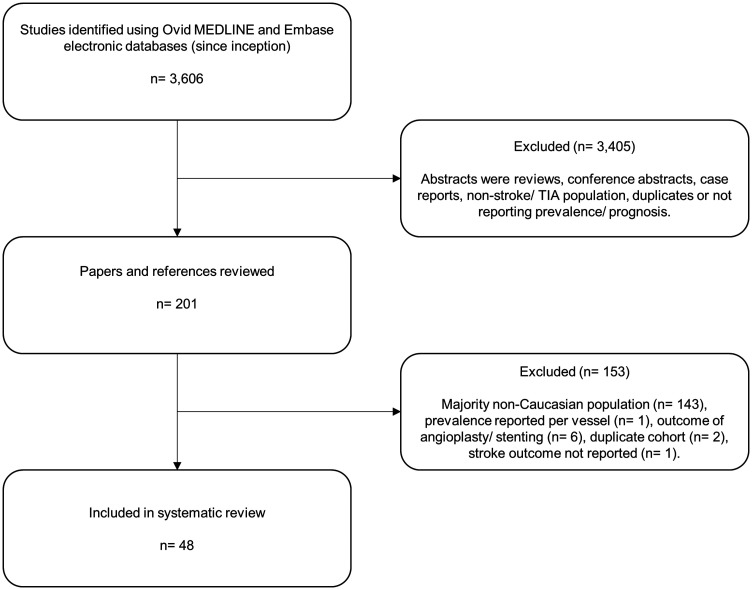
Flow diagram of systematic review article exclusions for ICS prevalence and prognosis in Caucasian TIA/stroke patients.

In this review, we highlight recent insights and advances into the prevalence, detection, prognosis, and treatment of symptomatic and asymptomatic ICS in Caucasian patients. Details of the literature search strategy and inclusion criteria are outlined in the Supplementary material, and a flow diagram of article exclusions is shown in Figure 1.

## Systematic review results

The systematic review identified 48 articles which fulfilled criteria studying ICS in Caucasian patients with ischemic stroke or transient ischemic attack (TIA) (Tables 1 and 2). Studies were hospital-based cohorts of consecutive ischemic stroke and TIA patients (39/81.3%: prospective *n* = 22, retrospective *n* = 17), two (4.2%) population-based studies of minor stroke TIA patients and the medical arms of clinical trials (*n* = 7/14.6%).

**Table 1.** Prevalence of intracranial stenosis in Caucasian TIA/stroke patients

Study	Location	Sample size	Mean age (years)	Caucasian (%)	IS/TIA	Definition of ICS		Prevalence of ICS		
						Screening imaging modality	Criteria	Any ICS *n* (%)	Asympt ICS *n* (%)	Sympt ICS *n* (%)
*All ICS*										
Sacco (NOMASS) 1995^w8^	USA	82	70	100	IS	TCD	Velocity criteria	–	–	*8* (1.0%)
Wityk 1996^w9^	USA	108	75	100	IS/TIA	TCD/MRA/CA	Velocity criteria or 50–100% stenosis	*26* (24.0)	–	*10* (9.0%)^a^
Thijs 2000^w13^	USA	1344	–	–	IS/TIA	CA/TCD/MRA	Velocity criteria or 50–99% stenosis	54 (4.0)	–	36 (2.7)
Weimar 2006^w20^	Germany	4157	67	–	IS/TIA	TCD/MRA/ CTA/CA	Velocity criteria or 50–100% stenosis	1259 (30.3)	–	611 (14.7)
Nahab (WASID)^b^ 2008^w22^	USA	312	∼64	50^b^	IS/TIA	CA/MRA	50–99% stenosis	–	79 (25.3%)	–
Holzer 2009^w23^	Germany	163	63	100	TIA	TCD	Velocity criteria	15 (9.2)	–	–
Meseguer 2010^w27^	France	1823	61	–	TIA	TCD	Velocity criteria	161 (8.8)	–	67 (3.7)
Weber 2010^w28^	Germany	13,584	67	–	IS/TIA	TCD/MRA	Velocity criteria or 50–99% stenosis	736 (5.4)	–	304 (2.2)
Homburg 2011^w30^	Netherlands	786	62	90	IS/TIA	CTA	50–100% stenosis	77 (9.8)	–	18 (2.3)
Von Weitzel-Mudersbach 2012^w31^	Denmark	195	66	100	TIA	TCD	Velocity criteria	24 (12.3)	–	16 (8.2)
Lau 2013^w32^	USA	539	66	83	IS/TIA	CTA	Any grade of stenosis	212 (39.3)	–	176 (32.7)
Ovesen 2013^w34^	Denmark	652	67	95	IS/TIA	CTA	≥30% stenosis	101 (15.5)	–	3 (0.5)
Ssi-Yan-Kai 2013^w36^	France	129	64	–	IS/TIA	MRA	50–100% stenosis	–	–	16 (12.4)
Wolff 2014^w37^	France	159	37	99	Young IS (age 18–45)	MRA/CTA/ DSA	≥50% stenosis	–	–	49 (31.2)
Logallo 2014^w38^	Norway	575	73	–	IS/TIA	TCD/MRA/ CTA	Velocity criteria or any degree stenosis	69 (12.0)	44 (7.7)	45 (7.8)
Tsivgoulis 2014^w39^	Greece	467	58	98	IS/TIA	TCD	Velocity criteria	51 (10.9)	9^c^ (1.9)	43 (9.2)
Gouveia 2014^w42^	Portugal	1302	72	–	IS/TIA	TCD/CTA	Velocity criteria or ≥50% stenosis	158 (12.1)	83 (6.3)	75 (5.8)
Baracchini 2016^w44^	Italy	1134	71	97	IS	TCD	Velocity criteria	–	–	99 (8.7)
Hoshino 2018^w46^	France	403	62	–	IS	TCD/MRA/ CTA	Velocity criteria or 50–100% stenosis	146 (36.2)	74^c^ (18.3)	72 (17.9)
Uchiyama 2019^w48^	France	3317	66	78	IS/TIA	TCD/MRA/ CTA	Velocity criteria or 50–100% stenosis	424 (12.8)	–	–
Hurford 2020^w49, w50^	UK	1368	62	94	Minor IS/TIA	MRA/CTA	50–100% stenosis	260 (19.0)	202 (14.8)	105 (7.7)
*Anterior circulation ICS only*										
Kappelle (NASCET)1999^w10^	USA	2589	66	95	IS/TIA	CA	50–100% ACA, MCA, intracranial ICA stenosis	–	–	14^d^ (0.5)
Baumgartner^e^ 2003^w15^	Switzerland	244	65	98	Lacunar stroke syndrome	TCD	Velocity criteria	30 (12.3)	–	16 (6.6%)
von Sarnowski^e^ 2013^w35^	Pan-European	1561	46	–	Young IS/TIA (age 18–55)	TCD	Velocity criteria	184 (11.8)	–	137 (8.8)
Mattioni 2014^w40^	Netherlands	220	65	–	IS/TIA	CTA	Intracranial ICA and MCA; any degree stenosis or occlusion	85 (38.6)	–	80 (36.4)
*Posterior circulation ICS only*										
Shin 1999^w12^	USA	430	60	88	Vertebrobasilar IS/TIA	CTA/MRA/CA	50–100% intracranial VA or BA stenosis	–	–	119 (27.7)
Baumgartner^e^ 2003^w15^	Switzerland	244	65	98	Lacunar stroke syndrome	TCD	Velocity criteria	19 (7.8)	–	13 (5.3)
Marquardt 2009^w24^	UK	141	69	95	Vertebrobasilar IS/TIA	MRA	≥50% intracranial VA or BA stenosis	–	–	14 (9.9)
Klein 2010^w26^	France	41	66	–	Pontine IS	MRA	≥30% BA stenosis	–	–	7 (17.1)
von Sarnowski^e^ 2013^w35^	Pan-European	1511	46	–	Young IS/TIA (age 18–55)	TCD	Velocity criteria	75 (5.0)	–	45 (3.0)

W: reference cited in supplementary material. Figures in italics derived from available data. IS: ischemic stroke; ICS: intracranial stenosis; CA: catheter angiography; TCD: transcranial Doppler; MRA: magnetic resonance angiography; CTA: computed tomography angiography; Asympt: asymptomatic; Sympt: symptomatic.

a Of entire cohort, but no racial/sex differences.

b WASID patients reviewed for coexistent asympt ICS; no significant racial differences in prevalence.

c Patients with asympt ICS reported but not how many with isolated asympt ICS or coexistent sympt ICS unknown.

d Ipsilateral to symptomatic ICA stenosis.

e Study reports anterior and posterior circulation ICS separately without noting duplicate patients hence are reported separately in this table.

**Table 2.** Prognosis of intracranial stenosis in medically-treated Caucasian TIA/stroke patients

Study	Location	Sample size	Mean age (years)	Caucasian (%)	IS/TIA	Definition of ICS		Prognosis of ICS		
						Screening imaging modality	Criteria	Mean follow-up (months)	Any IS *n* (%)	Same territory IS *n* (%)
*Symptomatic ICS*										
Hinton 1979^w2^	USA	16	62	–	IS/TIA	CA	40–100% MCA stenosis	36	–	2 (12.5)
EC/IC bypass study group 1985^w3^	Worldwide	714	56	–	IS/TIA	CA	Any degree of MCA and intracranial ICA stenosis	56	–	∼10.0% at 1 year
Wechler 1986^w4^	USA	12	62	–	IS/TIA	CA	50–100% carotid siphon stenosis	51	–	3 (25.0)
Moufarrijj 1986^w5^	USA	44	∼58	–	Vertebrobasilar IS/TIA	CA	≥50% intracranial VA or BA stenosis	73	–	5 (11.4)
Pessin 1987^w6^	USA	9	62	56	Vertebrobasilar IS/TIA	CA	≥40% distal BA stenosis	21	–	1 (11.1)
Pessin 1987^w7^	USA	6	70	–	PCA territory IS/TIA	CA	≥50% PCA stenosis	9	0	0
Kappelle (NASCET) 1999^w10^	USA	2589^a^	66	93	IS/TIA	CA	50-100% ACA, MCA, intracranial ICA stenosis	–	–	19.4–45.7% at 3 years
Woolfenden 1999^w11^	USA	26	72	82	Vertebrobasilar IS/TIA	CA/MRA	50–99% BA stenosis	23	5 (19.2)	4 (15.4)
Thijs 2000^w13^	USA	52	67	65	IS/TIA	CA/TCD/MRA	Velocity criteria or 50–99% stenosis	17	6 (11.5)	–
Arenillas 2001^w14^	Spain	40	62	–	IS/TIA	TCD	MCA velocity criteria	27	–	8 (20.0)
Qureshi 2003^w16^	USA	102	64	54	Vertebrobasilar IS/TIA	CA/MRA	50–99% intracranial VA or BA stenosis	15	14 (13.7)	8 (7.8)
Kern 2005^w18^	Germany	46	57	–	IS/TIA	TCD	Velocity criteria	31	15^b^ (32.6)	11^b^ (23.9)
Chimowitz (WASID) 2005^w19^	USA	280^c^	63	58	IS/TIA	TCD/MRA/ CTA	50–99% stenosis	22	57 (20.4)	42 (15.0)
Weimar 2006^w20^	Germany	272	67	–	IS/TIA	TCD/MRA/ CTA/CA	Velocity criteria or 50–100% stenosis	–	26 (9.6) at 1 year	–
Mazighi 2006^w21^	France	102	63	97	IS/TIA	MRA/CTA/CA	50–99% stenosis	23	–	14 (13.7)
Samaniego 2009^w25^	USA	58	65	83	IS/TIA	MRA/CTA/CA	Undefined	14	3 (5.1)	–
Weber 2010^w28^	Germany	197	65	–	IS/TIA	TCD/MRA	Velocity criteria or 50–99% stenosis	24	23.3% at 3 years	–
Kozak 2011^w29^	USA	25	61	72	IS/TIA	CA	50–99% stenosis	16	–	11 (44.0)
Nahab 2013^w33^	USA	22	66	59	IS/TIA	CA/CTA	50–99% stenosis	14	0	0
Ssi-Yan-Kai 2013^w36^	France	129	64	–	IS/ TIA	MRA	50–100% stenosis	–	0	0
Gouveia 2014^w42^	Portugal	72	73	–	IS/TIA	TCD/CTA	Velocity criteria or ≥50% stenosis	14	–	14 (19.4)
Derdeyn (SAMMPRIS) 2014^w41^	USA	227^a^	60	71	IS/TIA	CA	70–99% stenosis	32	–	31 (13.7)
Zaidat (VISSIT) 2015^w43^	USA	53^a^	62	72	IS/TIA	CA	70–99% stenosis	–	–	5 (9.4) at 1 year
Markus (VIST) 2017^w45^	UK	88^a^	67	–	Vertebrobasilar IS/TIA	MRA/CTA/CA	≥50% intracranial VA stenosis	29	–	4 (4.6)
Caliandro 2018^w47^	Italy	48	65	–	IS/TIA	TCD	Velocity criteria	–	–	5^c^ (10.4) at 1 year
Hoshino 2018^w46^	France	72	65	–	IS	TCD/MRA/CTA	Velocity criteria or 50–100% stenosis	–	9^b^ (13.2) at 4 years	–
Hurford 2020^w50^	UK	94	74	94	Minor IS/TIA	MRA/CTA	50–99% stenosis	29	12 (12.8)	8 (8.5)
*Asymptomatic ICS*										
Kremer 2004^w17^	Switzerland	53	67	–	IS/TIA	TCD	Velocity criteria	68	–	0
Kern 2005^w18^	Germany	56	66	–	IS/TIA	TCD	Velocity criteria	30	4^b^ (7.1)	2^b^ (3.6)
Nahab 2008^w22^	USA	100	66	50	IS/TIA	CA/MRA	50–99% stenosis	18	–	5 (5.0)
Gouveia 2014^w42^	Portugal	47	77	–	IS/TIA	TCD/CTA	Velocity criteria or ≥ 50% stenosis	14	–	1 (2.1)
Hoshino 2018^w46^	France	74	65	–	IS	TCD/MRA/CTA	Velocity criteria or 50–100% stenosis	–	8^b^ (11.5) at 4 years	–
Hurford 2020^w49^	UK	155	77	94	Minor IS/TIA	MRA/CTA	50–100% stenosis	40	8 (5.2)	5 (3.2)

W: reference cited in supplementary material. IS: ischemic stroke, ICS: intracranial stenosis; CA: catheter angiography; TCD: transcranial Doppler; MRA: magnetic resonance angiography; CTA: computed tomography angiography; MCA: middle cerebral artery; ICA: internal carotid artery; VA: vertebral artery; BA: basilar artery; PCA: posterior cerebral artery.

a Medical arm of trial.

b Ischemic stroke and TIA.

c Patients randomized to receive aspirin.

Of the 28 studies reporting ICS prevalence, 21 (75.0%) included all ICS, whereas seven studies (25.0%) only reported anterior or posterior circulation ICS (two studies reported both but did not identify duplicate patients so have been included twice in Table 1). Of the 29 studies reporting ICS prognosis, 23 (79.3%) reported the prognosis of symptomatic ICS only, 2 (6.9%) of asymptomatic ICS only, and 4 (13.8%) of both symptomatic and asymptomatic ICS (presented separately in Table 2). Seven studies (24.1%) did not report the mean follow-up time and were excluded from analyses of prognosis.

The study quality outcomes are shown in Supplementary Table 1; 24 studies (50.0%) were graded as fair, 14 poor (29.2%), and 10 good (20.8%) quality. The most frequent limitations were incomplete description of ICS definition, predominant use of TCD only, and lack of follow-up information.

## Definition and diagnosis of intracranial stenosis

**Figure 2.**
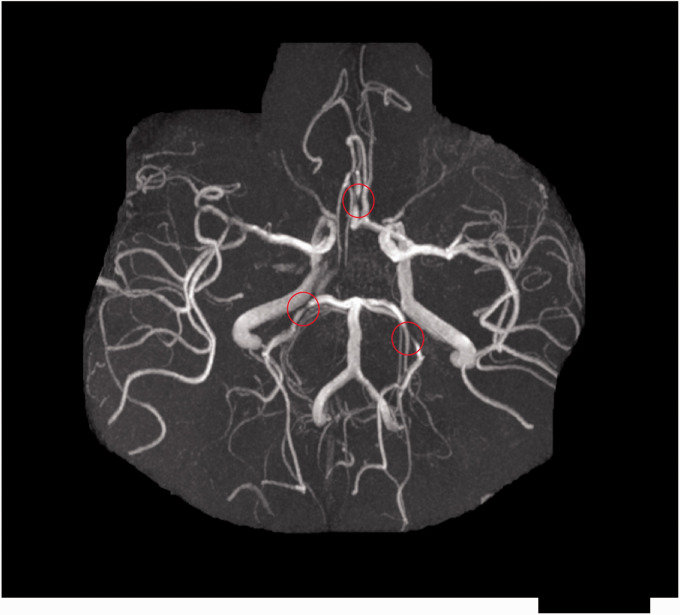
Time-of-flight MR angiogram of the large intracranial arteries showing multifocal atherosclerotic stenoses, including bilateral posterior cerebral arteries and right anterior cerebral artery (indicated by red circles).

ICS is a narrowing or occlusion of an intracranial (intradural or subarachnoid) arterial lumen due to atherosclerotic plaque (Figure 2). Atherosclerosis can be limited to the intracranial arteries or part of more systemic disease also affecting the coronary, renal, or peripheral arteries.^10^ It is important to distinguish non-atherosclerotic causes of intracranial vascular stenosis, including arterial dissection, moyamoya disease, intracranial vasculitis (idiopathic, infectious, or inflammatory), and vasospasm, as these conditions have different treatments and prognoses.^11^

There is variation between studies in the degree of luminal restriction and its method of measurement used to define ICS with cross-sectional angiography. The Warfarin–Aspirin Symptomatic Intracranial Disease (WASID) trial method^12^ compares the narrowest luminal diameter with the closest normal luminal diameter proximal to the stenosis (or distal if the proximal artery is also stenosed) and is most commonly used. Some investigators use a method analogous to the North American Symptomatic Carotid Endarterectomy Trial (NASCET), which considers the diameter of a site distal to the lesion as normal.^13^ Reliability of these methods has not been extensively investigated in ICS. One study of 25 patients with symptomatic middle cerebral artery (MCA) ICS found a significant difference in the degree of narrowing as determined by NASCET and WASID methodology on catheter angiography, but not CT angiography (CTA).^14^

In our systematic review, all studies using TCD defined ICS according to velocity parameters based on the Baumgartner criteria.^15^ In studies using cross-sectional angiography (*n* = 38), 20 studies (52.6%) calculated the degree of stenosis using the WASID trial method, 3 studies (7.9%) used criteria based on the NASCET, and 15 studies (39.5%) did not report the methodology used.

Greater degrees of luminal narrowing have been associated with higher risks of recurrent same-territory ischemic stroke.^16^ Consequently, although ≥50% stenosis is most commonly used in observational studies, randomized trials have typically recruited patients with 70–99% stenosis in order to enrich the study population.^9,17^ In our review, the degree of luminal narrowing used to define ICS with cross-sectional angiography (*n* = 38) was ≥50% in 27 studies (71.1%; 50–100% in 17, and 50–99% in 10), 70–99% in two studies (5.3%), ≥30% in two studies (5.3%), ≥40% in two studies (5.3%), any grade of luminal narrowing in four studies (10.6%), and unknown in one study (2.7%).

The gold standard imaging modality for detecting ICS is digital subtraction angiography (DSA) as it provides high-resolution visualization of the intracranial vasculature and, as a dynamic procedure, permits assessment of flow rates and direction in addition to assessment of collateral supply.^18^ However, DSA is invasive and is associated with a risk of serious complications in up to 1% of procedures and is therefore not appropriate for routine screening or research.^19,20^

Noninvasive angiography, such as transcranial Doppler ultrasound, MR angiography (MRA) or CTA, is safer, quicker, and more accessible in routine practice, but the available methods have differing sensitivities and specificities for ICS detection. Moreover, no single modality is suitable for all patients.

Transcranial Doppler ultrasound (TCD) identifies ICS by detecting increased flow velocity distal to the ICS. The Stroke Outcomes and Neuroimaging of Intracranial Atherosclerosis (SONIA) trial assessed the accuracy of TCD in detecting 50–99% ICS compared with DSA and reported negative and positive predictive values of 85% and 36%, respectively.^21^ Therefore, TCD can be a useful initial screening tool for ≥50–99% ICS but is limited to the major proximal intracranial arteries and to patients with adequate temporal acoustic bone windows. Also, there is some evidence the sensitivity and specificity of TCD is greater in the anterior than posterior circulation.^22^ TCD was the most commonly used modality identified in our systematic review; either as the sole modality (*n* = 12) or alongside cross-sectional angiography (*n* = 9).

CTA can detect and quantify ICS by opacification following administration of an iodine-based contrast, it is easily performed in routine practice, and can detect perfusion deficits when combined with CT-perfusion sequences. Studies comparing CTA with DSA for identification of ≥50% ICS have reported high sensitivities and specificities and a good inter-operator reliability.^13,23–25^ Patient-related limitations include the requirement for ionizing radiation and intravenous contrast. Technical limitations include the reduced spatial resolution of smaller intracranial vessels (particularly <2 mm),^26^ obscuration by extensive mural calcification,^27^ or susceptibility gradients, for example of the internal carotid artery near the sphenoid sinus.^26^ However, improving post-processing techniques mitigates many of these shortfalls.^28^

MRI can detect ICS either by time-of-flight (TOF) or contrast-enhanced sequences and has the advantage of offering detailed parenchymal imaging which may indicate the likely infarct mechanism. TOF-MRA does not use any radiation or contrast material to visualize the intracranial arteries and has variable sensitivity and specificity for ICS, but different magnet strengths and post-processing techniques have been used.^13,29,30^ One study comparing TOF-MRA and CTA in ICS detection concluded CTA was superior, with a higher sensitivity (98% vs. 70%) and positive predictive value (93% vs. 65%).^13^ The main limitation of TOF-MRA is the susceptibility to artifact because of flow abnormalities—low flow may mimic stenosis and turbulent or loss of laminar flow through stenosis may over- or underestimate its degree.^31–33^

Unlike TOF-MRA, gadolinium-based contrast enhanced MRA (CE-MRA) is not vulnerable to signal-intensity flow artifacts and can assess the origins of the major intracranial arteries. However, CE-MRA is more costly and increases the complexity of imaging, in particular requiring accurate timing of the contrast bolus, which is contraindicated in some patients.^34,35^ Older coil systems were limited by poor spatial resolution,^36^ but modern techniques have a similar sensitivity and specificity to TOF-MRA in detecting ICS.^33,37,38^

With the exception of TCD, the modalities discussed so far only allow diagnosis of ICS as defined by a degree of arterial luminal restriction, which may limit risk stratification.^39^ Although not yet widely adopted by clinical practice, novel post-processing techniques can be used to assess the downstream hemodynamic impact of an ICS, for example by noninvasive angiography to measure peri-stenotic flow by parameters such as fractional flow and translesional wall shear stress ratio.^39,40^ Similarly, modalities such as high-resolution MRI (HR-MRI) and intravascular ultrasonography, can provide direct assessment of plaque composition and detection of non-stenotic intracranial atheroma which may have clinical relevance.^41,42^ Recently symptomatic, unstable plaques have been shown to have a higher lipid content, intra-plaque hemorrhage and inflammatory cell infiltration,^43^ properties which can be detected by HR-MRI.^44^ Intravascular ultrasonography can detect fibrous, lipid, and calcific plaque constituents, but is rarely used as it is invasive and technically challenging.^45^

## Epidemiology of intracranial stenosis

### Intracranial stenosis in stroke/TIA patients

The importance of ICS as a cause of ischemic stroke in Asian, Black, and Hispanic populations is well recognized.^46^ The Northern Manhattan Stroke study has reported higher rates of ICS in Afro-Caribbean and Hispanic compared to Caucasian patients, with ICS attributed to 9% of strokes in Caucasians, 17% of African Americans, and 15% of Hispanics.^47^ Potential reasons for the differences seen in the prevalence of ICS between racial groups include genetic factors, such as ring finger protein 213 (RNF213)^48^ or salt sensitivity associated polymorphisms (e.g., α-adducin, angiotensinogen, and aldosterone synthase).^49^ There are also interracial differences in lifestyle and risk factor profiles,^50,51^ and due to a thinner media and adventitia and fewer elastic medial fibers compared to extracranial arteries, intracranial arteries are more vulnerable to hypertension-induced hemodynamic stress.^52^ Furthermore, ICS develops at younger ages in Asians than Caucasians with the reverse is seen with extracranial artery atherosclerosis^53^ and it has been postulated that protective antioxidant enzyme activity is greater in the intracranial arteries compared to the extracranial arteries at a younger age.^54^

Our review identified 28 studies of ICS prevalence in Caucasian stroke and TIA patients (Table 1). In these studies, 4166 of 34,563 patients (12.1%) were found to have any ICS, 2198 of 35,788 (6.4%) symptomatic ICS and 490 of 4427 (11.1%) asymptomatic ICS. There were significantly different rates of ICS in the pooled prospective (including trials and population-based studies) versus retrospective data: 12.2% versus 10.8% (*p* = 0.01) any ICS, and 5.8% versus 9.0% (*p* < 0.0001) symptomatic ICS.

**Figure 3.**
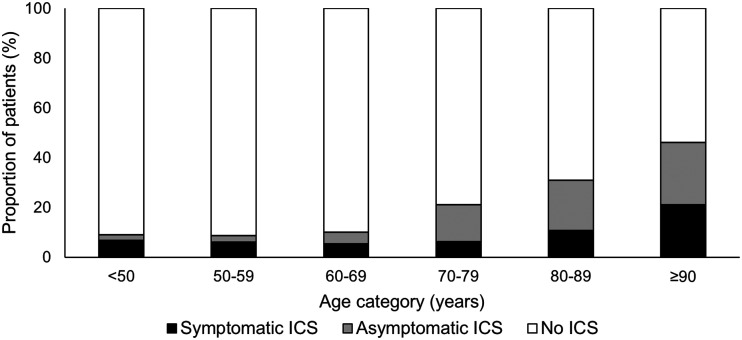
Age-specific prevalence of any symptomatic, only asymptomatic and no intracranial stenosis in minor ischemic stroke and TIA patients in the Oxford Vascular Study.

In an Oxford population-based study of 1368 Caucasian patients with TIA and minor ischemic stroke, 6.9% had symptomatic 50–99% ICS and this was heavily age dependent; increasing from 4.7% at <50 years to 19.6% at ≥90 years (Figure 3).^55^ In addition to being older, the patients with ICS had a higher burden of hypertension, diabetes mellitus, hyperlipidemia, atrial fibrillation, previous stroke, peripheral vascular disease, and ischemic heart disease.^55^

There have been few studies of the prevalence of asymptomatic, “incidental” ICS in a stroke and TIA population. In a post hoc analysis of WASID, coexistent asymptomatic ICS was identified in a quarter of participants,^56^ and in one hospital-based study of 403 stroke patients admitted to a single French center the asymptomatic ICS rate was 18.4%.^57^ In the aforementioned Oxford population-based study, 202 patients (14.8%) had any asymptomatic ICS similarly increasing with age; from 3.8% at <50 years to 34.6% at ≥90 years. Of note in this study asymptomatic ICS were more common than asymptomatic extracranial internal carotid artery disease. Older age, hypertension, and prior stroke/TIA were independent predictors of any asymptomatic ICS.^58^

### Intracranial stenosis in healthy participants

There are relatively few studies examining the prevalence of ICS in Caucasian patients without cerebrovascular disease. One large study of 1765 community-dwelling individuals estimated the US prevalence of ≥50% asymptomatic ICS for Caucasian 65–90 year olds as 8% using high-resolution MRA.^59^ The Barcelona-Asymptomatic Intracranial Atherosclerosis (AsIA) population-based study investigated 933 Spanish participants over the age of 50 years with transcranial color Doppler (TCCD) and reported a prevalence of moderate to severe ICS of 3.3%.^60^

## Prognosis of intracranial stenosis

### Mechanisms of stroke

The pathophysiology of infarction due to ICS is analogous to the mechanisms of extracranial internal carotid artery atherosclerosis-related infarction, and includes artery-to-artery embolism, in situ thrombo-occlusion, hypoperfusion due to subocclusive plaque and small perforating artery occlusion. The pattern of ischemia seen on neuroimaging can be suggestive of particular mechanisms. Border zone infarctions result from hypoperfusion due to a stenosed artery, territorial infarctions result from artery-to-artery embolism, and occlusion of small branching perforating arteries can cause subcortical strokes resembling lacunar infarcts.^61^ It is unclear whether the specific mechanism of ICS-related infarction has prognostic value, although in a post hoc analysis of the Stenting versus Aggressive Medical Therapy for Preventing Recurrent Stroke in Intracranial Stenosis (SAMMRPIS) trial data, patients with border zone infarctions were more likely to have poor collateral supply and were at the highest risk of recurrent stroke.^62^

### Prognosis of intracranial stenosis

Symptomatic ICS had been considered to convey a high risk of recurrent ischemic stroke; the SAMMPRIS trial sample size estimates were based on a primary endpoint rate of 29% at two years for comparable medically treated patients in WASID.^17^ The observed two-year primary endpoint rate in medically treated SAMMPRIS participants, 70% of who were Caucasian, was 14.1%, attributed to the more intensive secondary prevention therapy and lifestyle interventions.^8,63^

Our systematic review identified 29 studies that reported the prognosis of ICS in Caucasian minor stroke and TIA patients (Table 2). Of these, 19 (65.5%) reported the mean duration of patient follow-up and number of patients with recurrent ischemic stroke. In these studies, there were 89 (95% confidence interval (CI) = 74–108) any-territory ischemic strokes in 1000 person-years and 61 (95% CI = 52–71) same-territory ischemic strokes in 1000 person-years in patients with symptomatic ICS, and 10 (95% CI = 6–19) same-territory ischemic strokes in 1000 patient-years in patients with asymptomatic ICS.

There are few studies of ICS prognosis in Caucasian patients without cerebrovascular disease. One Spanish community-based cohort of 80 stroke-free participants with a high burden of vascular risk factors reported a rate of 2.9% and 12.6% of ischemic stroke and any vascular event/vascular death respectively during seven years follow-up.^64^ Intracranial carotid artery calcification volume (ICAC) was used as a surrogate marker of ICS in a sample population of the Rotterdam study, a population-based study of predominantly Caucasian community-dwelling individuals. The study included 2,323 stroke-free individuals of mean age 70 years; during 14,055 person-years of follow-up, 74 (3.2%) had an ischemic stroke and a larger ICAC volume was associated with a higher risk of stroke, independent of vascular risk factors.^65^

## Treatment of intracranial stenosis

### Medical management

Antiplatelet therapy is the principle antithrombotic treatment for patients with symptomatic ICS since the WASID trial demonstrated no benefit of warfarin over aspirin and higher rates of major hemorrhage in the warfarin arm.^66^ However, the role of direct oral anticoagulants has yet to be examined in patients with symptomatic ICS, and the combination of rivaroxaban and aspirin has been shown to reduce stroke risk in patients with systemic atherosclerosis.^67^

The optimal antiplatelet regime for treatment of recently symptomatic ICS has not been investigated by randomized trials. The Platelet-Oriented Inhibition in New TIA and Minor Ischemic Stroke (POINT) and Clopidogrel in High-Risk Patients with Acute Nondisabling Cerebrovascular Events (CHANCE) trials showed short-term dual antiplatelet therapy (DAPT) to be safe and effective in patients with high-risk TIA and minor ischemic stroke,^68,69^ and a subgroup analysis of CHANCE showed greater benefit in patients with symptomatic or asymptomatic ICS.^70^ A pooled analysis of these trials showed that the greatest benefits in stroke risk reduction were in the first 21 days,^71^ with longer term DAPT shown to increase the risk of major hemorrhage in the Management of Atherothrombosis with Clopidogrel in High-Risk Patients (MATCH)^72^ and Clopidogrel for High Atherothrombotic Risk and Ischemic Stabilization, Management, and Avoidance (CHARISMA)^73^ trials.

Short-term DAPT was used in the SAMMPRIS trial which reported a lower rate of recurrent ischemic stroke than expected based on the older WASID trial.^66^ Based on this, the American Heart Association/American Stroke Association (AHA/ASA) secondary stroke prevention guidelines state that treatment of recently (within 30 days) symptomatic 70–99% ICS with dual antiplatelet therapy for 90 days might be reasonable.^74^ However, the independent contribution of the antiplatelet regimen is unclear as the SAMMPRIS treatment protocol also included intensive risk factor management and lifestyle advice.^63^

Alternative antiplatelet agents, such as ticagrelor or prasugrel, may be more efficacious in patients with symptomatic atherosclerosis, particularly in cases of clopidogrel resistance. In a subgroup analysis of the Acute Stroke or Transient Ischemic Attack Treated with Aspirin or Ticagrelor and Patient Outcomes (SOCRATES) trial, ticagrelor was superior to aspirin in prevention of vascular events or death 90 days in patients with acute ischemic stroke or TIA due to ipsilateral extra- or ICS.^75^

Management of the primary risk factors for atherosclerosis (elevated blood pressure, poor glycemic control, and elevated low-density lipoprotein (LDL) cholesterol) has been shown to be effective secondary prevention of ischemic stroke of any etiology.^76,77^ Evidence in patients with symptomatic ICS is derived indirectly from randomized trials. Risk factor management in WASID was not standardized, but subgroup analysis revealed improved outcomes in patients with a mean systolic blood pressure <140 mmHg,^78^ total mean cholesterol < 200 mg/dL^79^ and HbA1c of <7%.^80^ These findings were the basis of the intensive medical management protocol of SAMMPRIS, which aimed for systolic blood pressure <140 mmHg (or <130 mmHg in patients with diabetes mellitus), an LDL cholesterol level <70 mg/dL (1.81 mmol/L) and HbA1c of <7%.^63^ The Treat Stroke to Target (TST) trial recently confirmed this LDL target to be more effective at reducing recurrent vascular events than patients with a target of 90–110 mg/dL (2.3–2.8 mmol/L).^81^ In addition, SAMMPRIS employed a lifestyle modification program for increased physical activity, optimized nutrition, and weight loss and smoking cessation advice.^80^

There are no randomized trials informing the management of asymptomatic or remotely symptomatic ICS. As described previously, they can be a common finding in older patients with cerebrovascular disease and in those with vascular risk factors.^58,60^ The risk of recurrent ischemic stroke in patients found to have incidental, asymptomatic ICS is low, and management should follow standard secondary prevention guidelines.^82^

### Endovascular therapy

Until the Food and Drug Administration approved the self-expanding Wingspan stent (Stryker Neurovascular) for treatment of recently symptomatic 50–99% ICS, there had only been published case series demonstrating high periprocedural complication rates.^83^ SAMMPRIS commenced shortly after the approval and randomized patients with 70–99% recently (within 30 days) symptomatic ICS (TIA or minor ischemic stroke) to percutaneous transluminal angioplasty and stenting (PTAS) with the Wingspan stent and intensive medical management or intensive medical management alone.^17^

Recruitment to SAMMPRIS was stopped early due to a significantly higher rate of post-procedure stroke (due to perforating vessel occlusion) or death; 14% versus 6% in the non-stenting arm.^17^ Post hoc analyses concluded that the higher degrees of ICS and earlier treatment windows (compared to the previous Wingspan registries), but not operator experience, may have increased this periprocedural risk.^84,85^ Furthermore, an old infarct in the territory of the ICS on baseline imaging, a new stroke presentation, and the absence of statin use at enrollment were independently associated with a high risk of recurrent stroke.^86^ There were no risk differences between Caucasian and Black patients or other subgroups in a preplanned sensitivity analysis.^87^

The Vitesse Intracranial Stent Study for Ischemic Stroke Therapy (VISSIT) trial started shortly after SAMMPRIS and had a similar protocol and patient mix (70% Caucasian), with the exception of investigating the PHAROS Vitesse balloon-expandable stent (Codman Neurovascular). As with SAMMPRIS, VISSIT was stopped early as the 30-day rate of ischemic stroke or TIA was higher in the intervention arm (24.1% vs. 9.4%), and at one year, 36.2% in the stent group had a stroke or TIA, versus 15.1% in the non-stenting group.^9^

**Figure 4.**
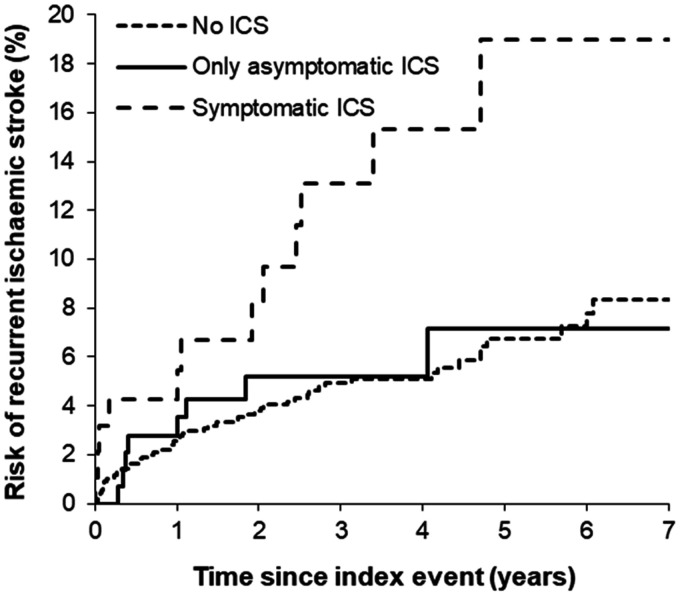
Kaplan–Meier graph showing the seven-year risks of recurrent ischemic stroke in minor ischemic stroke/ TIA patients with 50–99% symptomatic, asymptomatic, or no intracranial stenosis in the Oxford Vascular Study.

Aside from the high procedural risks, SAMMPRIS and VISST were criticized for the lower than expected rates of recurrent stroke in the non-stenting arms and the relatively young cohort (mean age < 60 years). However, a validation study of symptomatic ICS prognosis in an older, population-based TIA and minor stroke cohort confirmed a low rate of recurrent stroke on intensively treated medical patients (Figure 4; one-year risk of recurrent ischemic stroke 5.6%).^55^

Patients with recently symptomatic posterior circulation ICS are at particularly high risk of early recurrent stroke.^88^ The Vertebral Artery Ischaemia Stenting Trial (VIST) sought to compare vertebral artery (VA) PTAS and medical management with medical treatment alone for recently symptomatic extra- or intracranial VA stenosis, but was stopped after 182 participants because of slow recruitment. Although underpowered, there were no significant differences in outcome between arms in patients with intracranial VA stenosis, but overall a nonsignificant 60% lower risk of recurrent stroke in the PTAS arm during a median follow-up of 3.5 years, driven by fewer complications in the extracranial VA stenting group.^89^

Although the Wingspan Stent System Post Market Surveillance (WEAVE) trial, has shown an improved periprocedural complication rate with Wingspan stents,^90^ current AHA/ASA guidelines do not recommend PTAS for patients with symptomatic ICS even if the event occurred while receiving antiplatelet therapy. For patients with recurrent events despite optimal medical treatment, the benefit of PTAS is unclear and should be considered investigational.^82^

Improved patient selection may improve the safety and efficacy of PTAS in symptomatic ICS. The ongoing China Angioplasty and Stenting for Symptomatic Intracranial Severe Stenosis (CASSISS) trial is comparing best medical therapy with/without PTAS in patients with 70–99% symptomatic ICS. The investigators exclude patients with perforator stroke without MRI appearances of distal hypoperfusion or artery-to-artery embolism and delay stenting for three weeks following the index event in order to reduce periprocedural risks.^91^

### Surgical therapy

The Extracranial to Intracranial (EC/IC) Bypass Study was an international, randomized controlled trial which failed to show the superiority of arterial bypass (superficial temporal artery to the MCA) and medical therapy over medical therapy alone in patients with extracranial carotid occlusion, intracranial carotid, or MCA stenosis.^92^ The procedure is no longer routinely performed for symptomatic atherosclerotic ICS but the indirect revascularization technique, encephaloduroarteriosynangiosis (EDAS), is of emerging interest.^93^

## Conclusion and future directions

ICS is a highly prevalent cause of stroke worldwide with important ethnic disparities. ICS has previously been neglected as a cause of stroke in Caucasians but is highly prevalent at older ages and frequently discovered with the growing use of non-invasive angiography. Intensive medical therapy, including antiplatelet medication, risk factor control, and lifestyle advice, is the treatment of choice. However, a subgroup of patients with ICS experience recurrent ischemic stroke despite medical therapy. Future research should aim at establishing standard approaches to detecting ICS, elucidating the ethnic differences in risk and developing biomarkers to identify high-risk patients. Furthermore, there is a need to develop novel treatments or therapeutic approaches to lower the risk of stroke in these higher risk patients.

## References

[1] Kolominsky-Rabas PL Weber M Gefeller O Neundoerfer B Heuschmann PU. Epidemiology of ischemic stroke subtypes according to TOAST criteria: incidence, recurrence, and long-term survival in ischemic stroke subtypes: a population-based study. Stroke 2001; 32: 2735–2740. 11739965 10.1161/hs1201.100209

[2] Sacco RL Kargman DE Gu Q Zamanillo MC. Race-ethnicity and determinants of intracranial atherosclerotic cerebral infarction. The Northern Manhattan Stroke Study. Stroke 1995; 26: 14–20. 7839388 10.1161/01.str.26.1.14

[3] Wityk RJ Lehman D Klag M Coresh J Ahn H Litt B. Race and sex differences in the distribution of cerebral atherosclerosis. Stroke 1996; 27: 1974–1980. 8898801 10.1161/01.str.27.11.1974

[4] Huang YN Gao S Li SW, et al. Vascular lesions in Chinese patients with transient ischemic attacks. Neurology 1997; 48: 524–525. 9040750 10.1212/wnl.48.2.524

[5] Liu HM Tu YK Yip PK Su CT. Evaluation of intracranial and extracranial carotid steno-occlusive diseases in Taiwan Chinese patients with MR angiography: preliminary experience. Stroke 1996; 27: 650–653. 8614924 10.1161/01.str.27.4.650

[6] Wong KS Li H. Long-term mortality and recurrent stroke risk among Chinese stroke patients with predominant intracranial atherosclerosis. Stroke 2003; 34: 2361–2366. 12947158 10.1161/01.STR.0000089017.90037.7A

[7] Suwanwela NC Chutinetr A. Risk factors for atherosclerosis of cervicocerebral arteries: intracranial versus extracranial. Neuroepidemiology 2003; 22: 37–40. 12566952 10.1159/000067112

[8] Derdeyn CP Chimowitz MI Lynn MJ, et al. Aggressive medical treatment with or without stenting in high-risk patients with intracranial artery stenosis (SAMMPRIS): the final results of a randomised trial. Lancet 2014; 383: 333–341. 24168957 10.1016/S0140-6736(13)62038-3 PMC3971471

[9] Zaidat OO Fitzsimmons B-F Woodward BK, et al. Effect of a balloon-expandable intracranial stent vs medical therapy on risk of stroke in patients with symptomatic intracranial stenosis: the VISSIT randomized clinical trial. JAMA 2015; 313: 1240–1248. 25803346 10.1001/jama.2015.1693

[10] Qureshi AI Caplan LR. Intracranial atherosclerosis. Lancet 2014; 383: 984–998. http://www.sciencedirect.com/science/article/pii/S0140673613610880. 24007975 10.1016/S0140-6736(13)61088-0

[11] Bang OY Toyoda K Arenillas JF Liu L Kim JS. Intracranial large artery disease of non-atherosclerotic origin: recent progress and clinical implications. J Stroke 2018; 20: 208–217. 29886713 10.5853/jos.2018.00150 PMC6007295

[12] Samuels OB Joseph GJ Lynn MJ Smith HA Chimowitz MI. A standardized method for measuring intracranial arterial stenosis. AJNR Am J Neuroradiol 2000; 21: 643–646. 10782772 PMC7976653

[13] Bash S Villablanca JP Jahan R, et al. Intracranial vascular stenosis and occlusive disease: evaluation with CT angiography, MR angiography, and digital subtraction angiography. AJNR Am J Neuroradiol 2005; 26: 1012–1021. 15891154 PMC8158600

[14] Huang J Degnan AJ Liu Q, et al. Comparison of NASCET and WASID criteria for the measurement of intracranial stenosis using digital subtraction and computed tomography angiography of the middle cerebral artery. J Neuroradiol 2012; 39: 342–345. 22197402 10.1016/j.neurad.2011.11.005

[15] Baumgartner RW Mattle HP Schroth G. Assessment of >/=50% and <50% intracranial stenoses by transcranial color-coded duplex sonography. Stroke 1999; 30: 87–92. 9880394 10.1161/01.str.30.1.87

[16] Kasner SE Chimowitz MI Lynn MJ, et al. Predictors of ischemic stroke in the territory of a symptomatic intracranial arterial stenosis. Circulation 2006; 113: 555–563. 16432056 10.1161/CIRCULATIONAHA.105.578229

[17] Chimowitz MI Lynn MJ Derdeyn CP, et al. Stenting versus aggressive medical therapy for intracranial arterial stenosis. N Engl J Med 2011; 365: 993–1003. 21899409 10.1056/NEJMoa1105335 PMC3552515

[18] Barr JD. Cerebral angiography in the assessment of acute cerebral ischemia: guidelines and recommendations. J Vasc Interv Radiol 2004; 15: S57–S66. 15101516 10.1097/01.rvi.0000107491.61085.59

[19] Willinsky RA Taylor SM TerBrugge K Farb RI Tomlinson G Montanera W. Neurologic complications of cerebral angiography: prospective analysis of 2,899 procedures and review of the literature. Radiology 2003; 227: 522–528. 12637677 10.1148/radiol.2272012071

[20] Kaufmann TJ Huston 3rd J Mandrekar JN Schleck CD Thielen KR Kallmes DF. Complications of diagnostic cerebral angiography: evaluation of 19,826 consecutive patients. Radiology 2007; 243: 812–819. 17517935 10.1148/radiol.2433060536

[21] Mturi N Alcock K Carter JA Newton CR Lange JH LaPorte RE Talbott EO Chang YF Monsurrò MR Aiello I Morgante L. Stroke outcome and neuroimaging of intracranial atherosclerosis (SONIA): design of a prospective, multicenter trial of diagnostic tests. Neuroepidemiology 2004; 23: 23–32. 14765534 10.1159/000073971

[22] Feldmann E Wilterdink JL Kosinski A, et al. The Stroke Outcomes and Neuroimaging of Intracranial Atherosclerosis (SONIA) trial. Neurology 2007; 68: 2099–2106. 17409371 10.1212/01.wnl.0000261488.05906.c1

[23] Nguyen-Huynh MN Wintermark M English J, et al. How accurate is CT angiography in evaluating intracranial atherosclerotic disease?. Stroke 2008; 39: 1184–1188. 18292376 10.1161/STROKEAHA.107.502906

[24] Roubec M Kuliha M Jonszta T, et al. Detection of intracranial arterial stenosis using transcranial color-coded duplex sonography, computed tomographic angiography, and digital subtraction angiography. J Ultrasound Med 2011; 30: 1069–1075. 21795482 10.7863/jum.2011.30.8.1069

[25] Duffis EJ Jethwa P Gupta G Bonello K Gandhi CD Prestigiacomo CJ. Accuracy of computed tomographic angiography compared to digital subtraction angiography in the diagnosis of intracranial stenosis and its impact on clinical decision-making. J Stroke Cerebrovasc Dis 2013; 22: 1013–1017. 22464276 10.1016/j.jstrokecerebrovasdis.2012.02.016

[26] Skutta B Fürst G Eilers J Ferbert A Kuhn FP. Intracranial stenoocclusive disease: double-detector helical CT angiography versus digital subtraction angiography. AJNR Am J Neuroradiol 1999; 20: 791–799. 10369348 PMC7056155

[27] Marquering HA Nederkoorn PJ Bleeker L van den Berg R Majoie CB. Intracranial carotid artery disease in patients with recent neurological symptoms: high prevalence on CTA. Neuroradiology 2013; 55: 179–185. 23053000 10.1007/s00234-012-1097-6

[28] Saba L Sanfilippo R Montisci R Mallarini G. Assessment of intracranial arterial stenosis with multidetector row CT angiography: a postprocessing techniques comparison. AJNR Am J Neuroradiol 2010; 31: 874–879. 20053812 10.3174/ajnr.A1976 PMC7964195

[29] Hirai T Korogi Y Ono K, et al. Prospective evaluation of suspected stenoocclusive disease of the intracranial artery: combined MR angiography and CT angiography compared with digital subtraction angiography. AJNR Am J Neuroradiol 2002; 23: 93–101. 11827880 PMC7975497

[30] Choi CG Lee DH Lee JH, et al. Detection of intracranial atherosclerotic steno-occlusive disease with 3D time-of-flight magnetic resonance angiography with sensitivity encoding at 3T. AJNR Am J Neuroradiol 2007; 28: 439–446. 17353309 PMC7977826

[31] Korogi Y Takahashi M Mabuchi N, et al. Intracranial vascular stenosis and occlusion: diagnostic accuracy of three-dimensional, Fourier transform, time-of-flight MR angiography. Radiology 1994; 193: 187–193. 8090890 10.1148/radiology.193.1.8090890

[32] Heiserman JE Drayer BP Keller PJ Fram EK. Intracranial vascular stenosis and occlusion: evaluation with three-dimensional time-of-flight MR angiography. Radiology 1992; 185: 667–673. 1438743 10.1148/radiology.185.3.1438743

[33] Nederkoorn PJ Elgersma OEH Mali WPTM Eikelboom BC Kappelle LJ van der Graaf Y. Overestimation of carotid artery stenosis with magnetic resonance angiography compared with digital subtraction angiography. J Vasc Surg 2002; 36: 806–813. 12368742

[34] Leclerc X Gauvrit JY Nicol L Pruvo JP. Contrast-enhanced MR angiography of the craniocervical vessels: a review. Neuroradiology 1999; 41: 867–874. 10639659 10.1007/s002340050858

[35] Yang CW Carr JC Futterer SF, et al. Contrast-enhanced MR angiography of the carotid and vertebrobasilar circulations. AJNR Am J Neuroradiol 2005; 26: 2095–2101. 16155164 PMC8148843

[36] van den Wijngaard IR Holswilder G van Walderveen MAA, et al. Treatment and imaging of intracranial atherosclerotic stenosis: current perspectives and future directions. Brain Behav 2016; 6: e00536. 27843693 10.1002/brb3.536 PMC5102638

[37] Wutke R Lang W Fellner C, et al. High-resolution, contrast-enhanced magnetic resonance angiography with elliptical centric k-space ordering of supra-aortic arteries compared with selective X-ray angiography. Stroke 2002; 33: 1522–1529. 12052985 10.1161/01.str.0000016972.70366.d6

[38] Willinek WA von Falkenhausen M Born M, et al. Noninvasive detection of steno-occlusive disease of the supra-aortic arteries with three-dimensional contrast-enhanced magnetic resonance angiography: a prospective, intra-individual comparative analysis with digital subtraction angiography. Stroke 2005; 36: 38–43. 15569881 10.1161/01.STR.0000149616.41312.00

[39] Liebeskind DS. Understanding blood flow: the other side of an acute arterial occlusion. Int J Stroke 2007; 2: 118–120. 18705965 10.1111/j.1747-4949.2007.00117.x

[40] Liebeskind DS Feldmann E. Fractional flow in cerebrovascular disorders. Interv Neurol 2013; 1: 87–99. 23730308 10.1159/000346803 PMC3666952

[41] Arenillas JF. Intracranial atherosclerosis: current concepts. Stroke 2011; 42: S20–S23. 21164126 10.1161/STROKEAHA.110.597278

[42] Klein IF Lavallee PC Mazighi M Schouman-Claeys E Labreuche J Amarenco P. Basilar artery atherosclerotic plaques in paramedian and lacunar pontine infarctions: a high-resolution MRI study. Stroke 2010; 41: 1405–1409. 20538696 10.1161/STROKEAHA.110.583534

[43] Chen XY Wong KS Lam WWM Zhao H-L Ng HK. Middle cerebral artery atherosclerosis: histological comparison between plaques associated with and not associated with infarct in a postmortem study. Cerebrovasc Dis 2008; 25: 74–80. 18033961 10.1159/000111525

[44] Turan TN Bonilha L Morgan PS Adams RJ Chimowitz MI. Intraplaque hemorrhage in symptomatic intracranial atherosclerotic disease. J Neuroimag 2011; 21: e159–e161. 10.1111/j.1552-6569.2009.00442.x 19909397

[45] Diethrich EB Pauliina Margolis M Reid DB, et al. Virtual histology intravascular ultrasound assessment of carotid artery disease: the Carotid Artery Plaque Virtual Histology Evaluation (CAPITAL) study. J Endovasc Ther Off J Int Soc Endovasc Spec 2007; 14: 676–686. 10.1177/152660280701400512 17924734

[46] Wong LKS. Global burden of intracranial atherosclerosis. Int J Stroke 2006; 1: 158–159. 18706036 10.1111/j.1747-4949.2006.00045.x

[47] White H Boden-Albala B Wang C, et al. Ischemic stroke subtype incidence among whites, blacks, and Hispanics: the Northern Manhattan Study. Circulation 2005; 111: 1327–1331. 15769776 10.1161/01.CIR.0000157736.19739.D0

[48] Liao X Zhang T Li B, et al. Rare RNF213 variants and the risk of intracranial artery stenosis/occlusion disease in Chinese population: a case-control study. BMC Med Genet 2019; 20: 55. 30925911 10.1186/s12881-019-0788-9 PMC6441181

[49] Kokubo Y. Prevention of hypertension and cardiovascular diseases: a comparison of lifestyle factors in Westerners and East Asians. Hypertension 2014; 63: 655–660. 24420548 10.1161/HYPERTENSIONAHA.113.00543

[50] Kim JS Bonovich D. Research on intracranial atherosclerosis from the East and west: why are the results different?. J Stroke 2014; 16: 105–113. 25328869 10.5853/jos.2014.16.3.105 PMC4200588

[51] Forouhi NG Sattar N. CVD risk factors and ethnicity—a homogeneous relationship?. Atheroscler Suppl 2006; 7: 11–19. 10.1016/j.atherosclerosissup.2006.01.003 16500156

[52] Ritz K Denswil NP Stam OCG van Lieshout JJ Daemen MJAP. Cause and mechanisms of intracranial atherosclerosis. Circulation 2014; 130: 1407–1414. 25311618 10.1161/CIRCULATIONAHA.114.011147

[53] Kim JS Kim Y-J Ahn S-H Kim BJ. Location of cerebral atherosclerosis: why is there a difference between East and West?. Int J Stroke 2018; 13: 35–46. 27145795 10.1177/1747493016647736

[54] D'Armiento FP Bianchi A de Nigris F, et al. Age-related effects on atherogenesis and scavenger enzymes of intracranial and extracranial arteries in men without classic risk factors for atherosclerosis. Stroke 2001; 32: 2472–2479. 11692003 10.1161/hs1101.098520

[55] Hurford R Wolters FJ Li L Lau KK Küker W Rothwell PM. Prevalence, predictors, and prognosis of symptomatic intracranial stenosis in patients with transient ischaemic attack or minor stroke: a population-based cohort study. Lancet Neurol 2020; 19: 413–421. 32333899 10.1016/S1474-4422(20)30079-X PMC7116132

[56] Nahab F Cotsonis G Lynn M, et al. Prevalence and prognosis of coexistent asymptomatic intracranial stenosis. Stroke 2008; 39: 1039–1041. 18239161 10.1161/STROKEAHA.107.499475 PMC3506394

[57] Hoshino T Sissani L Labreuche J, et al. Prevalence of systemic atherosclerosis burdens and overlapping stroke etiologies and their associations with long-term vascular prognosis in stroke with intracranial atherosclerotic disease. JAMA Neurol 2018; 75: 203–211. 29279888 10.1001/jamaneurol.2017.3960 PMC5838618

[58] Hurford R Wolters FJ Li L Lau KK Küker W Rothwell PM. Prognosis of asymptomatic intracranial stenosis in patients with transient ischemic attack and minor stroke. JAMA Neurol 2020; 77: 947–954. 32453401 10.1001/jamaneurol.2020.1326 PMC7251503

[59] Suri MFK Qiao Y Ma X, et al. Prevalence of intracranial atherosclerotic stenosis using high-resolution magnetic resonance angiography in the general population: the atherosclerosis risk in communities study. Stroke 2016; 47: 1187–1193. 27056984 10.1161/STROKEAHA.115.011292 PMC5319392

[60] Lopez-Cancio E Dorado L Millan M, et al. The Barcelona-asymptomatic intracranial atherosclerosis (AsIA) study: prevalence and risk factors. Atherosclerosis 2012; 221: 221–225. 22245037 10.1016/j.atherosclerosis.2011.12.020

[61] Feng X Chan KL Lan L, et al. Stroke mechanisms in symptomatic intracranial atherosclerotic disease: classification and clinical implications. Stroke 2019; 50: 2692–2699. 31409268 10.1161/STROKEAHA.119.025732

[62] Wabnitz AM Derdeyn CP Fiorella DJ, et al. Hemodynamic markers in the anterior circulation as predictors of recurrent stroke in patients with intracranial stenosis. Stroke 2018. STROKEAHA118020840. 10.1161/STROKEAHA.118.020840 PMC6559874 30580705

[63] Chaturvedi S Turan TN Lynn MJ, et al. Do patient characteristics explain the differences in outcome between medically treated patients in SAMMPRIS and WASID?. Stroke 2015; 46: 2562–2567. 26251251 10.1161/STROKEAHA.115.009656 PMC4550543

[64] Planas-Ballve A Crespo AM Aguilar LM, et al. The Barcelona-asymptomatic intracranial atherosclerosis study: subclinical intracranial atherosclerosis as predictor of long-term vascular events. Atherosclerosis 2019; 282: 132–136. 30731285 10.1016/j.atherosclerosis.2019.01.022

[65] Bos D Portegies MLP van der Lugt A, et al. Intracranial carotid artery atherosclerosis and the risk of stroke in whites: the Rotterdam study. JAMA Neurol 2014; 71: 405–411. 24535643 10.1001/jamaneurol.2013.6223

[66] Chimowitz MI Lynn MJ Howlett-Smith H, et al. Comparison of warfarin and aspirin for symptomatic intracranial arterial stenosis. N Engl J Med 2005; 352: 1305–1316. 15800226 10.1056/NEJMoa043033

[67] Sharma M Hart RG Connolly SJ, et al. Stroke outcomes in the COMPASS trial. Circulation 2019; 139: 1134–1145. 30667279 10.1161/CIRCULATIONAHA.118.035864

[68] Wang Y Wang Y Zhao X, et al. Clopidogrel with aspirin in acute minor stroke or transient ischemic attack. N Engl J Med 2013; 369: 11–19. 23803136 10.1056/NEJMoa1215340

[69] Johnston SC Easton JD Farrant M, et al. Clopidogrel and aspirin in acute ischemic stroke and high-risk TIA. N Engl J Med 2018; 379: 215–225. 29766750 10.1056/NEJMoa1800410 PMC6193486

[70] Liu L Wong KSL Leng X, et al. Dual antiplatelet therapy in stroke and ICAS: subgroup analysis of CHANCE. Neurology 2015; 85: 1154–1162. 26330567 10.1212/WNL.0000000000001972 PMC4603889

[71] Pan Y Elm JJ Li H, et al. Outcomes associated with clopidogrel-aspirin use in minor stroke or transient ischemic attack: a pooled analysis of clopidogrel in high-risk patients with acute non-disabling cerebrovascular events (CHANCE) and platelet-oriented inhibition in new TIA and minor ischemic stroke (POINT) trials. JAMA Neurol 2019; 76: 1466–1473. 10.1001/jamaneurol.2019.2531 PMC6704730 31424481

[72] Diener H-C Bogousslavsky J Brass LM, et al. Aspirin and clopidogrel compared with clopidogrel alone after recent ischaemic stroke or transient ischaemic attack in high-risk patients (MATCH): randomised, double-blind, placebo-controlled trial. Lancet 2004; 364: 331–337. 15276392 10.1016/S0140-6736(04)16721-4

[73] Bhatt DL Fox KAA Hacke W, et al. Clopidogrel and aspirin versus aspirin alone for the prevention of atherothrombotic events. N Engl J Med 2006; 354: 1706–1717. 16531616 10.1056/NEJMoa060989

[74] Kernan WN Ovbiagele B Black HR, et al. Guidelines for the prevention of stroke in patients with stroke and transient ischemic attack: a guideline for healthcare professionals from the American Heart Association/American Stroke Association. Stroke 2014; 45: 2160–2236. 24788967 10.1161/STR.0000000000000024

[75] Amarenco P Albers GW Denison H, et al. Efficacy and safety of ticagrelor versus aspirin in acute stroke or transient ischaemic attack of atherosclerotic origin: a subgroup analysis of SOCRATES, a randomised, double-blind, controlled trial. Lancet Neurol 2017; 16: 301–310. 28238711 10.1016/S1474-4422(17)30038-8

[76] PROGRESS Collaborative Group. Randomised trial of a perindopril-based blood-pressure-lowering regimen among 6,105 individuals with previous stroke or transient ischaemic attack. Lancet 2001; 358: 1033–1041. . 11589932 10.1016/S0140-6736(01)06178-5

[77] Amarenco P Bogousslavsky J Callahan A 3rd, et al. High-dose atorvastatin after stroke or transient ischemic attack. N Engl J Med 2006; 355: 549–559. 16899775 10.1056/NEJMoa061894

[78] Turan TN Cotsonis G Lynn MJ Chaturvedi S Chimowitz M. Relationship between blood pressure and stroke recurrence in patients with intracranial arterial stenosis. Circulation 2007; 115: 2969–2975. 17515467 10.1161/CIRCULATIONAHA.106.622464

[79] Chaturvedi S Turan TN Lynn MJ, et al. Risk factor status and vascular events in patients with symptomatic intracranial stenosis. Neurology 2007; 69: 2063–2068. 18040012 10.1212/01.wnl.0000279338.18776.26

[80] Turan TN Lynn MJ Nizam A, et al. Rationale, design, and implementation of aggressive risk factor management in the Stenting and Aggressive Medical Management for Prevention of Recurrent Stroke in Intracranial Stenosis (SAMMPRIS) trial. Circ Cardiovasc Qual Outcomes 2012; 5: e51–e60. 22991350 10.1161/CIRCOUTCOMES.112.966911 PMC3500085

[81] Amarenco P Kim JS Labreuche J, et al. A comparison of two LDL cholesterol targets after ischemic stroke. N Engl J Med 2020; 382: 9. 31738483 10.1056/NEJMoa1910355

[82] Kernan WN Ovbiagele B Black HR, et al. Guidelines for the prevention of stroke in patients with stroke and transient ischemic attack. Stroke 2014; 45: 2160–2236. 24788967 10.1161/STR.0000000000000024

[83] Cruz-Flores S Diamond AL. Angioplasty for intracranial artery stenosis. Cochrane Database Syst Rev 2006, pp. CD004133. 16856032 10.1002/14651858.CD004133.pub2 PMC8764997

[84] Derdeyn CP Fiorella D Lynn MJ, et al. Impact of operator and site experience on outcomes after angioplasty and stenting in the SAMMPRIS trial. J Neurointerv Surg 2013; 5: 528–533. 22977278 10.1136/neurintsurg-2012-010504 PMC3652908

[85] Banerjee C Chimowitz MI. Stroke caused by atherosclerosis of the major intracranial arteries. Circ Res 2017; 120: 502–513. 28154100 10.1161/CIRCRESAHA.116.308441 PMC5312775

[86] Waters MF Hoh BL Lynn MJ, et al. Factors associated with recurrent ischemic stroke in the medical group of the SAMMPRIS trial. JAMA Neurol 2016; 73: 308–315. 26747792 10.1001/jamaneurol.2015.4315 PMC5576955

[87] Lutsep HL Lynn MJ Cotsonis GA, et al. Does the stenting versus aggressive medical therapy trial support stenting for subgroups with intracranial stenosis?. Stroke 2015; 46: 3282–3284. 26382173 10.1161/STROKEAHA.115.009846 PMC4624506

[88] Gulli G Marquardt L Rothwell PM Markus HS. Stroke risk after posterior circulation stroke/transient ischemic attack and its relationship to site of vertebrobasilar stenosis: pooled data analysis from prospective studies. Stroke 2013; 44: 598–604. 23386676 10.1161/STROKEAHA.112.669929

[89] Markus HS Larsson SC Kuker W, et al. VIST Investigators. Stenting for symptomatic vertebral artery stenosis: the Vertebral Artery Ischaemia Stenting Trial. Neurology 2017; 89: 1229–1236. 28835400 10.1212/WNL.0000000000004385 PMC5606920

[90] Alexander MJ Zauner A Chaloupka JC, et al. WEAVE trial: final results in 152 on-label patients. Stroke 2019; 50: 889–894. 31125298 10.1161/STROKEAHA.118.023996

[91] Gao P Zhao Z Wang D, et al. China Angioplasty and Stenting for Symptomatic Intracranial Severe Stenosis (CASSISS): a new, prospective, multicenter, randomized controlled trial in China. Interv Neuroradiol 2015; 21: 196–204. 25934656 10.1177/1591019915581778 PMC4757239

[92] EC/IC Bypass Study Group*. Failure of extracranial-intracranial arterial bypass to reduce the risk of ischemic stroke. Results of an international randomized trial. N Engl J Med 1985; 313: 1191–1200. . 2865674 10.1056/NEJM198511073131904

[93] Gonzalez NR Dusick JR Connolly M, et al. Encephaloduroarteriosynangiosis for adult intracranial arterial steno-occlusive disease: long-term single-center experience with 107 operations. J Neurosurg 2015; 123: 654–661. 26067617 10.3171/2014.10.JNS141426

